# Long-Term Outcome of HBV-Infected Patients with Clinically Significant Portal Hypertension Achieving Viral Suppression

**DOI:** 10.3390/jpm12020239

**Published:** 2022-02-08

**Authors:** Mathias Jachs, Lukas Hartl, David Bauer, Benedikt Simbrunner, Albert Friedrich Stättermayer, Robert Strassl, Michael Trauner, Mattias Mandorfer, Thomas Reiberger

**Affiliations:** 1 Department of Internal Medicine III, Division of Gastroenterology and Hepatology, Medical University of Vienna, A-1090 Vienna, Austria; mathias.jachs@meduniwien.ac.at (M.J.); lukas.a.hartl@meduniwien.ac.at (L.H.); david.bauer@meduniwien.ac.at (D.B.); benedikt.simbrunner@meduniwien.ac.at (B.S.); albertfriedrich.staettermayer@meduniwien.ac.at (A.F.S.); michael.trauner@meduniwien.ac.at (M.T.); mattias.mandorfer@meduniwien.ac.at (M.M.); 2 Vienna Hepatic Hemodynamic Laboratory, Division of Gastroenterology and Hepatology, Medical University of Vienna, A-1090 Vienna, Austria; 3 Christian Doppler Laboratory for Portal Hypertension and Liver Fibrosis, Medical University of Vienna, A-1090 Vienna, Austria; 4 Department of Laboratory Medicine, Division of Clinical Virology, Medical University of Vienna, A-1090 Vienna, Austria; robert.strassl@meduniwien.ac.at

**Keywords:** cirrhosis, portal hypertension, antivirals, nucleos(t)ide analogs, disease regression, transient elastography, liver stiffness

## Abstract

Background: Nucleos(t)ide analog (NA) treatment for hepatitis B virus (HBV) infection may improve clinically significant portal hypertension (CSPH). Data on hepatic venous pressure gradient (HVPG) and non-invasive tests (NITs) for risk re-stratification in virally suppressed HBV-infected patients with pre-treatment CSPH are limited. Methods: We retrospectively included patients with long-term (>12 months) suppression of HBV replication and pre-treatment CSPH (i.e., varices, collaterals on cross-sectional imaging, or ascites). Patients were monitored by on-treatment liver stiffness measurement (LSM) and HVPG assessment. The primary outcome was (further) hepatic decompensation (including liver-related mortality). Results: Forty-two patients (*n* = 12 (28.6%) with previous decompensation, HBeAg-negative: *n* = 36 (85.7%)) were included and followed for 2.1 (0.6; 5.3) years. The median HVPG (available in *n* = 17) was 15 (10; 22) mmHg and the median LSM 22.5 (12.5; 41.0) kPa. LSM correlated strongly with HVPG (Spearman’s ρ: 0.725, *p* < 0.001) and moderately with the model for end-stage liver disease (MELD) score (ρ: 0.459, *p* = 0.002). LSM, MELD and albumin levels had good prognostic value for decompensation (area under the receiver operated characteristics curve (AUROC) >0.850 for all). LSM predicted (further) decompensation in competing risk regression (subdistribution hazard ratio (SHR): 1.05 (95% confidence interval(CI) 1.03–1.06); *p* < 0.001), even after adjusting for other factors. An LSM cut-off at 25kPa accurately stratified patients into a low-risk (*n* = 23, zero events during follow-up) and a high-risk (*n* = 19; *n* = 12 (63.2%) developed events during follow-up) group. Conclusions: Patients with HBV-induced CSPH who achieved long-term viral suppression were protected from decompensation, if LSM was <25 kPa. LSM ≥ 25 kPa indicates a persisting risk for decompensation, despite long-term HBV suppression.

## 1. Introduction

Nucleos(t)ide analog (NA) treatment is recommended for all patients with hepatitis B virus (HBV)-induced cirrhosis with detectable HBV-DNA [1,2]. Importantly, long-term NA treatment achieves viral suppression in the vast majority patients [3,4], while HBsAg loss [5] as a biomarker of functional cure is rarely achieved.

Studies have demonstrated beneficial effects of NA therapy in patients with cirrhosis/advanced chronic liver disease (ACLD) such as liver fibrosis regression [6], hepatocellular carcinoma (HCC) prevention [7] and protection from liver-related events, i.e., hepatic decompensation, and mortality [8]. However, the impact of NA therapy on clinically significant portal hypertension (CSPH) and disease dynamics upon suppression remain less well defined. In a small uncontrolled trial involving 19 HBV patients with cirrhosis and CSPH, i.e., a hepatic venous pressure gradient (HVPG) ≥10 mmHg, one year of lamivudine (3TC) therapy induced a decrease in HVPG in all but one patient, who had experienced viral breakthrough [9]. In another observational study investigating the effects of different NA (including 3TC, adefovir and tenofovir disoproxilfumarate (TDF)), patients without varices at baseline rarely developed varices during follow-up, and patients who had already developed small varices prior to NA therapy showed regression in a vast majority of cases (67%) [10]. Importantly, the worsening of CSPH, i.e., growth/progression of varices almost exclusively occurred in the context of HCC development in this study, and the risk-modifying effects of HCC diagnosis on the progression of portal hypertension in HBV-infection warrant further investigation.

In contrast to HBV, the evolution of CSPH after antiviral therapy has been extensively investigated in hepatitis C (HCV) following antiviral therapy [11,12]. Furthermore, NITs have been developed for ruling-in/ruling-out CSPH after antiviral therapy in HCV [13,14], and the use of liver stiffness measurement (LSM) via vibration-controlled transient elastography (VCTE) and platelet count (PLT) for risk stratification in patients who achieved an HCV-cure is now recommended in clinical practice [15].

Despite the large body of evidence for beneficial effects of NA-induced HBV suppression, ACLD-specific information remains scarce. Moreover, no study has investigated the correlation between HVPG and NITs in virally suppressed patients or their use for re-stratification of risk in HBV-induced ACLD patients who are known to have had CSPH pre therapy. Thus, we aimed to investigate (i) the correlation between NITs and HVPG and (ii) the prognostic value of NITs in HBV patients with pre-treatment CSPH achieving virologic suppression during long-term NA therapy.

## 2. Materials and Methods

In this retrospective analysis, we included patients with HBV-induced advanced chronic liver disease (ACLD) who (i) were diagnosed with chronic HBV-infection at our center between 2007 and 2020, (ii) were under long-term (>1 year) stable NA therapy at the time of study inclusion (i.e., on-treatment LSM), and (iii) had pre-treatment CSPH as evident from varices, a history of variceal bleeding, collaterals on cross-sectional imaging, ascites, and/or pre-treatment HVPG ≥10 mmHg and (iv) underwent an on-treatment follow-up LSM examination with our without HVPG measurement.

Patients who had a history of (i) hepatocellular carcinoma (HCC), (ii) transjugular intrahepatic portosystemic shunt (TIPS) or (iii) liver transplantation were excluded.

Information on events indicating (further) hepatic decompensation, HCC, liver transplantation, or death occurring after study inclusion were recorded.

HVPG-measurements were conducted following a standardized operating procedure described elsewhere [16]. Laboratory tests were performed at the ISO-certified Department of Laboratory Medicine of the Medical University of Vienna. LSM were conducted using the FibroScan^®^ (Echosens, Paris, France) system.

A history of variceal bleeding or past/current ascites/hepatic encephalopathy defined dACLD.

Clinical events during follow-up (FU) that were considered as hepatic decompensation in compensated patients comprised variceal bleeding, development of overt hepatic encephalopathy (HE), development of ascites and liver-related death. Further decompensation in patients who had already developed decompensation at baseline was defined as variceal bleeding, requirement of paracentesis, grade 3/4 HE or liver-related death. The primary outcome of this study was the incidence of first/further decompensation in the overall patient cohort.

Statistical analysis was conducted using R 4.0.2 (R Core Team, R Foundation for Statistical Computing, Vienna, Austria) and GraphPad Prism 8 (GraphPad Software, La Jolla, CA, USA). Categorial variables are presented as number and percentages of patients with the respective characteristic. Continuous variables are presented as median (25th; 75th percentile). Group comparisons were conducted by Fisher’s exact test or Wilcoxon rank-sum test as applicable. Correlations were investigated by calculating Spearman’s ρ.

Patients entered our time-to-event analysis models at the time of on-treatment LSM and were followed until the time of (further) decompensation, HCC development, liver transplantation or death. The area under the receiver operating characteristic curve (AUROC) for decompensation within the first three years of follow-up was analyzed for all investigated NITs using the r package timeROC [17], taking into account competing risks, i.e., HCC development, liver transplantation and non-liver-related death; the optimal LSM cut-off for prognostication was derived from Youden’s index. Cumulative incidences for (further) decompensation were calculated and compared between groups using Gray’s test, and prognostic models were developed by applying competing risk regression as proposed by Fine and Gray [18]. In all models, HCC development, liver transplantation and non-liver-related death were considered as competing risks. Univariate models incorporated variables that differed significantly between patients who developed decompensation during FU versus those who did not. Significant variables were carried on to the multivariate analyses, and the final model was restricted to two variables owing to the low incidence of events in this cohort. LSM as the NIT with the highest AUROC was adjusted separately for all other variables, resulting in three models (A–C). Variable selection was evaluated by stepwise regression analysis with backward elimination incorporating all variables that were carried on from univariate analysis, showing that model (A) had the lowest prediction error as estimated by Akaike information criterion (AIC). A two-sided *p*-value < 0.05 was considered statistically significant.

This study was conducted in accordance with the Declaration of Helsinki and approved by the Institutional Review Board (IRB) of the Medical University of Vienna (No. 1515/2020, date of approval: 30 June 2020). No written informed consent was required for this retrospective analysis.

## 3. Results

### 3.1. Patient/Treatment Characteristics

Forty-two patients with HBV-induced pre-treatment CPSH were included. Detailed information on patient/treatment characteristics is provided in the Supplementary Materials (Table S1). As per inclusion criteria, on-treatment LSM was available in all patients, whereas HVPG measurements were conducted in *n* = 17 (40.5%) patients.

The median time between NA-therapy initiation and follow-up characterization was 2.5 (1.2; 4.9) years. All patients were virally suppressed by NA therapy with no viral breakthroughs.

Twelve (28.6%) patients had a history of hepatic decompensation. Metabolic cofactors were common: alcohol consumption (above 30 g/d for men and 20 g/d for women) and diabetes mellitus were present in 21.4% and 28.6% of patients, respectively.

### 3.2. Correlation of HVPG with VCTE-LSM and Biomarkers of Disease Severity

**Figure 1:**
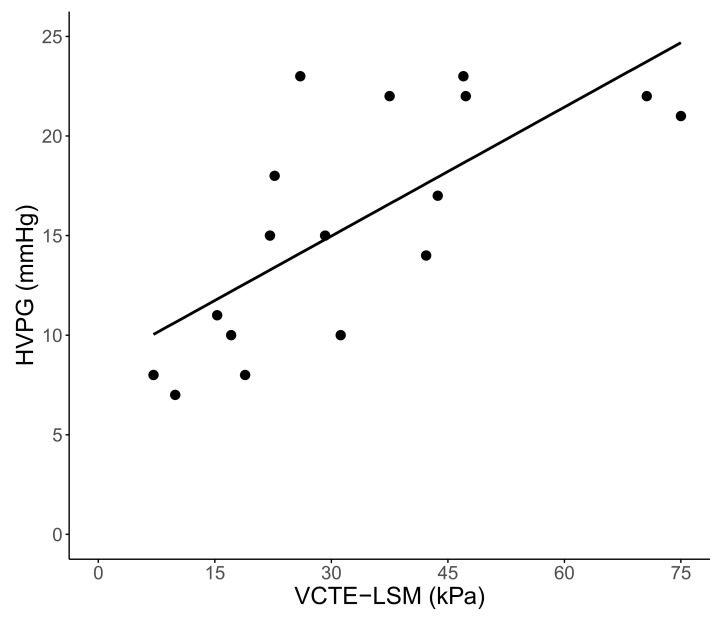
Correlation of the hepatic venous pressure gradient (HVPG) and liver stiffness measurement (LSM) attained by vibration-controlled transient elastography (VCTE) (*n* = 17, ρ = 0.725, *p* < 0.001).

LSM and HVPG correlated strongly (ρ: 0.725, *p* < 0.001) in the *n* = 17 patients who also underwent HVPG measurement, as shown in Figure 1. In contrast, HVPG did not correlate with PLT (ρ: −0.117, *p* = 0.655), nor with MELD (ρ: 0.048, *p* = 0.856). In the overall cohort, correlations of moderate strength were found between LSM and MELD (ρ: 0.459, *p* = 0.002), and LSM and PLT (ρ: −0.474, *p* = 0.001).

### 3.3. Clinical FU

Patients were followed for a median of 2.1 (0.6; 5.3) years. Twelve (28.6%) patients developed one or more events of (further) decompensation: Development/worsening of ascites was seen in eight, development/worsening of HE in seven, and variceal (re-)bleeding in two patients. Notably, decompensation was preceded by an HBV-DNA flare (without ALT-elevation) in one patient, while no other HBV-DNA-/ALT-flares associated with decompensation were observed in our cohort during follow-up. HCC was diagnosed in 6 (14.3%) patients. Four (9.5%) patients underwent liver transplantation. Finally, nine (21.4%) and four (9.5%) patients died of liver-related and non-liver-related causes, respectively.

### 3.4. Comparison of Characteristics between Patients with or without (Further) Decompensation during FU

Patients who showed (further) decompensation during FU were more likely to have a history of previous decompensation, most importantly ascites (58.3% versus 13.3% of patients without decompensation during FU, *p* = 0.008), as shown in Table 1. Accordingly, they had more advanced disease, i.e., higher MELD (11 (10; 12) versus 8 (7; 10) points, *p* = 0.002) and lower levels of serum albumin (34.6 (29.8; 36.3) versus 39.8 (37.5; 43.7) g/L, *p* = 0.001). Regarding portal hypertension, HVPG (available in *n* = 17) was higher (22 (21; 22) versus 12 (9; 16) mmHg, *p* = 0.023) in patients with decompensation during FU. Similarly, LSM was considerably higher in the latter group of patients (47.7 (31.7; 75.0) versus 16.3 (10.0; 24.2) kPa, *p* < 0.001) who also had lower PLT levels (63 (48; 119) versus 146 (91; 195) G/L, *p* = 0.010). The metabolic profile (BMI, prevalence of diabetes and alcohol intake) was similar between the two groups.

Notably, patients with decompensation during FU were characterized significantly earlier than patients who remained compensated during FU (time from NA initiation to LSM/HVPG-measurement: 1.1 (1.1; 1.7) versus 4.3 (2.2; 5.3) years, *p* = 0.002).

### 3.5. Prognostic Value of Patient Characteristics for (Further) Decompensation during FU

**Figure 2:**
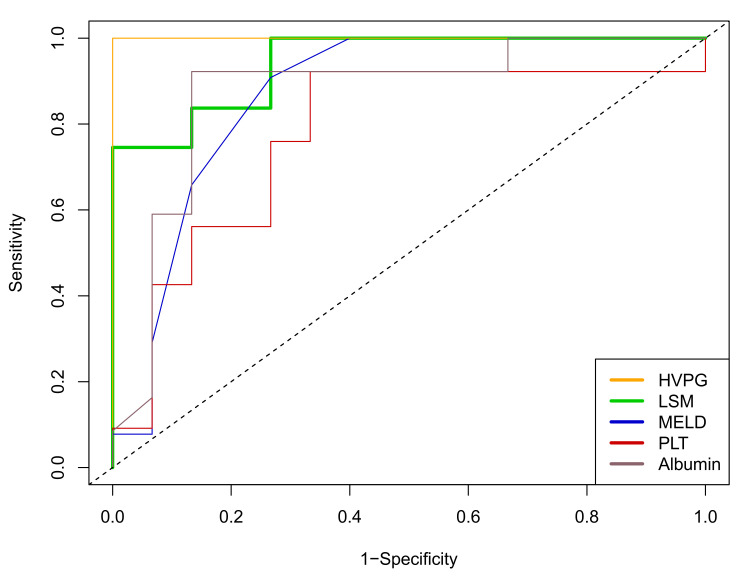
Time-dependent receiver operating characteristic curves for the prediction of (further) hepatic decompensation within three years of follow-up by HVPG (AUROC = 1.000 (95%CI 1.000–1.000); *n* = 17), LSM (0.944 (0.868–1.000)), MELD (0.869 (0.729–1.000)), PLT (0.775 (0.586–0.964), inverse association) and albumin levels (0.873 (0.727–1.000), inverse association). Abbreviations: HVPG = hepatic venous pressure gradient; LSM = liver stiffness measurement; MELD = model for end-stage liver disease score; PLT = platelet count; AUROC = area under the receiver operating characteristics curve.

In the cohort of patients who underwent HVPG measurements (*n* = 17), HVPG values had an area under the receiver operated characteristics curve (AUROC) of 1.000 (95% CI: 1.000–1.000) for the prediction of (further) decompensation during follow-up. In the overall cohort, LSM, MELD, and albumin levels showed robust prognostic performances (AUROC for all: >0.850), while PLT was less predictive, as shown in Figure 2.

In univariate competing risk regression models considering (further) decompensation as the outcome of interest and HCC development, liver transplantation and non-liver-related mortality as competing risks (liver transplantation was always preceded by further decompensation or HCC development), LSM values, (a history of or current) ascites and MELD score (all *p* < 0.001) as well as serum albumin levels (*p* = 0.002) had strong prognostic implications, while PLT count was of no prognostic value in this context (*p* = 0.140), as shown in Table S2.

Owing to the low (*n* = 12) number of events, multivariate models were restricted to two variables. As determined by stepwise regression using backward elimination (Table S3), the best model was Model comprised LSM with an adjusted subdistribution hazard ratio (aSHR) of 1.03 per kPa (95% confidence interval (95%CI): 1.01–1.06, *p* = 0.006) and ascites (aSHR: 3.43 (95%CI: 0.93–12.63). *p* = 0.064). LSM showed prognostic value that was also independent from all other markers that were carried on from univariate analysis, as shown in Table 2 as Models (A–C).

The ideal cut-off for prognostication of decompensation during FU via LSM as estimated by Youden’s index was 26.0 kPa; however, we applied a cut-off of 25 kPa (as recently proposed for ruling-in CSPH by the unpublished Baveno VII Consensus) that also showed 100% negative predictive value for decompensation during FU.

### 3.6. FU Data Stratified by VCTE-LSM ≥ 25 kPa versus <25 kPa

**Figure 3:**
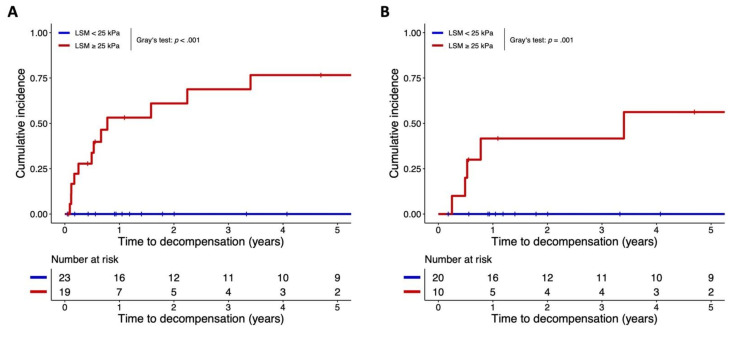
Cumulative incidence of (**A**) (further) hepatic decompensation in the overall cohort and of (**B**) first hepatic decompensation in patients with compensated cirrhosis, stratified by liver stiffness ≥ 25 kPa versus < 25 kPa at follow-up examination. Importantly, HCC diagnosis, liver transplantation and non-liver related mortality were considered as competing risks (graphs not shown). Abbreviations: LSM = liver stiffness measurement; HCC = hepatocellular carcinoma.

Decompensation during FU occurred exclusively in the 19 patients with LSM ≥ 25 kPa (*n* = 12, 63.2%), while the 23 patients with liver stiffness < 25 kPa were protected from decompensation (*n* = 0, 0.0%); the respective cumulative incidences are shown in Figure 3 (Gray’s test: *p* < 0.001). Four (21.1%) and two (8.7%) of patients with LSM ≥ 25 or <25 kPa developed HCC during FU, respectively. Liver transplantation was performed in four (21.1%) patients with ≥25 kPa, while none of the patients with lower liver stiffness underwent transplantation. Finally, liver-related death occurred in nine (47.4%) of the patients with high LSM values (zero non-liver-related deaths were observed), while zero (0%) liver-related and four (17.4%) non-liver-related deaths were recorded in the patients with VCTE-LSM < 25 kPa.

### 3.7. Prognostication in Patients with Compensated Advanced Chronic Liver Disease (cACLD)

In the subgroup of patients with cACLD at the time of on-treatment LSM (*n* = 30), LSM (and other NITs) conferred similar prognostic value in AUROC analysis and competing risk regression in comparison to the overall cohort, as shown in Table S4 and Figure S1. Only univariate analyses were conducted, owing to the low number (*n* = 5) of events in the cACLD cohort. Again, events were exclusively recorded in patients that surpassed the ≥25 kPa threshold (*n* = 5 (50.0%) developed first decompensation during FU), as compared to zero events in the <25 kPa group (Gray’s test: *p* = 0.001); the cumulative incidences are shown in Figure 3.

### 3.8. Patients with Additional LSM Values

**Figure 4:**
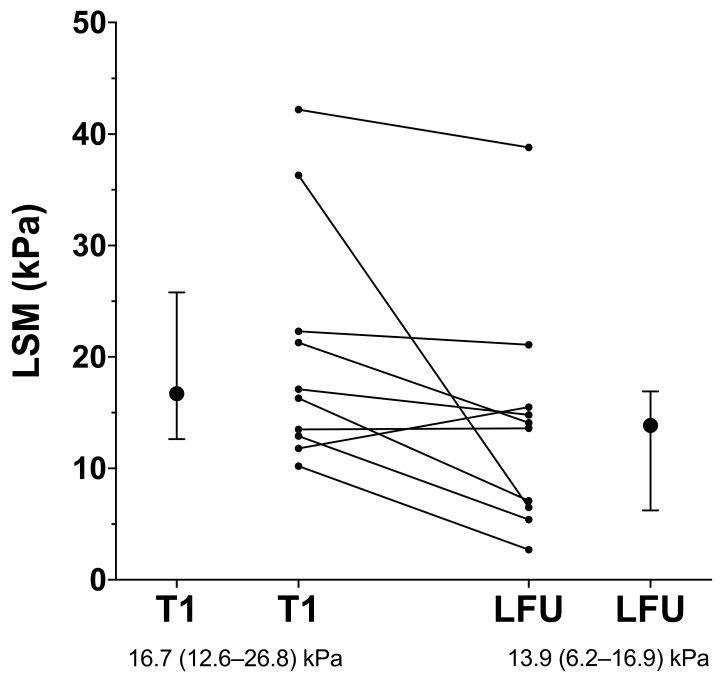
Changes in liver stiffness over time (presented as individual paired values and median and IQR) in patients undergoing an additional liver stiffness measurement during long-term follow-up (LFU). Only one patient showed an increase; the patient was diagnosed with HCC soon after. Abbreviations: T1 = first measurement LSM = liver stiffness measurement; HCC = hepatocellular carcinoma.

Ten (23.8%) patients underwent additional LSM, with the last examination occurring after a median of 2.9 (1.2; 5.5) years of FU. Except for one patient, who was diagnosed with HCC shortly afterwards, all patients showed further decreases in liver stiffness, as shown in Figure 4. Notably, the decreases in LSM values observed in nine patients were accompanied by in improvement in MELD (7 (6; 8) points during follow-up versus 8 (7; 11) points at baseline; *p* = 0.087), and two patients regressed from Child-B to Child-A cirrhosis, while the other patients already had Child-A cirrhosis at baseline and remained stable.

## 4. Discussion

In our cohort of 42 thoroughly characterized cirrhotic HBV patients with pre-treatment CSPH, broadly available NITs—most importantly LSM—performed upon long-term stable viral suppression accurately predicted risk for decompensation, including liver-related mortality. LSM showed an excellent correlation with HVPG, whilst also being weakly associated with liver dysfunction, i.e., MELD, and PLT levels.

Most of the investigated NITs (LSM, MELD, and albumin levels) performed well in AUROC analyses for the prediction of decompensation during long-term HBV suppression, except for PLT count. Notably, the prognostic value of HVPG was excellent in the subgroup of patients who underwent hepatic vein catheterization, indicating the crucial pathomechanistic role of portal hypertension for decompensation in HBV patients with suppressed viral replication, which complements findings from HCV patients after direct acting antiviral (DAA) therapy [11,19].

The high cumulative incidence of (further) hepatic decompensation of 28.6% under chronic HBV suppression in our cohort is explained by the fact that patients who underwent liver vein catheterization had HVPG values that were mostly well above the CSPH threshold of ≥10 mmHg, with almost 50% surpassing the 16 mmHg threshold indicating severe portal hypertension [20], and some patients already experienced decompensation events prior to NA treatment initiation. Overall, patients who developed (further) decompensation during FU had worse liver function, i.e., higher MELD, and more severe portal hypertension, i.e., higher HVPG and lower PLT, all of which correlated with LSM.

In competing risk regression, on-treatment LSM had prognostic value for hepatic decompensation during FU in analyses adjusted for either ascites, MELD or albumin.

Importantly, an LSM cut-off at 25 kPa (derived from Youden’s index and recently proposed by the unpublished Baveno VII Consensus for ruling-in CSPH after etiological cure) accurately assigned patients to a low-risk group (without hepatic decompensation during FU, negative predictive value: 100%) and a high-risk group for hepatic decompensation despite NA-induced HBV suppression. Notably, HCC occurred in both groups, highlighting the need for maintaining HCC screening in cirrhotic patients with pre-treatment CSPH who achieve HBV suppression.

Next, the findings regarding the prognostic performance of the investigated NITs were confirmed in a sensitivity analysis restricted to the patients with cACLD, suggesting that the NITs are more than mere reflections of the underlying disease status and—after further validation—may be applied in clinical practice as prognostic markers.

Lastly, our observation of regressing risk for decompensation in patients with NA-induced suppression of HBV and pretreatment CSPH was confirmed in a subset of patients who underwent an additional (‘last’) LSM during long-term FU. Except for patients developing HCC, continued decreases in liver stiffness were seen in all HBV-infected patients under NA-induced virologic suppression.

A major strength of our study is the validation of the correlation of NITs with the diagnostic gold standard for prognostication in portal hypertension, i.e., invasively measured HVPG. Interestingly, LSM conferred a similar prognostic value to HVPG. Importantly, we did not evaluate the prognostic value of pre-treatment levels of NITs; however, it was demonstrated in HCV patients after antiviral therapy that (standalone) post-treatment levels of NITs more accurately predict outcome [14]. Patients with pre-therapy CSPH that show high LSM levels despite long-term NA-induced suppression of HBV should thus be carefully monitored, and preventive treatment, most importantly non-selective betablockers (NSBB), should be initiated/maintained to prevent decompensation [21,22], also due to recently described beneficial non-hemodynamic effects on systemic inflammation [23,24] that has been identified as a key driver of decompensation in advanced cirrhosis [25].

Some limitations of this study need to be addressed: First, the low sample size and the unstandardized timepoints of follow-up LSM limit the conclusions that can be drawn from our study. However, strong signals were observed despite the low number of patients, which argues for the strength of the reported associations. Second, HVPG measurements were only conducted in a subgroup of patients. Those patients were generally more advanced, which may be explained by referral bias because clinicians are more likely to refer patients to an invasive re-evaluation if complications occur or liver function deteriorates despite etiological control. This also explains why only three out of 17 patients who underwent hepatic vein catheterization showed on-treatment HVPG levels below 10 mmHg as definite proof of resolution of CSPH induced by NA. However, LSM showed a strong correlation with (i) HVPG and (ii) outcome, and thus it may be assumed that a high proportion of the 54.8% of patients with LSM < 25 kPa (and thus protection from deleterious outcomes) had in fact resolved CSPH. Third, no definite conclusions regarding causality may be drawn from this retrospective analysis.

## 5. Conclusions

In summary, our study demonstrated that on-treatment LSM is a valuable, readily available tool for risk stratification in HBV-infected patients with pre-treatment CSPH and NA-induced viral suppression. LSM correlates well with portal hypertension in suppressed HBV patients with cirrhosis and CSPH, and an LSM cut-off at <25 kPa identified patients who were protected from decompensation or liver-related mortality during long-term FU. Patients that fail to improve to LSM < 25 kPa need close surveillance, and NSBB-treatment of portal hypertension, preferably with carvedilol [21].

## Figures and Tables

**Table 1:** Comparison of patient characteristics under long-term chronic NA therapy stratified by the incidence of hepatic decompensation during FU.

Patient Characteristics	No Hepatic Decompensation during FU *n* = 30	Hepatic Decompensation during FU *n* = 12	*p*-Value
Sex, male/female (% male)	23/7 (76.7%)	10/2 (83.3%)	0.953
Age, years (IQR)	46.5 (40.1; 56.4)	50.3 (47.7; 56.5)	0.242
BMI, kg/m^2^	26.6 (22.4; 30.9)	24.6 (23.6; 27.5)	0.452
HBeAg positive (%)	5 (16.7%)	1 (12.5%)	1.000
NA compound			0.453
TDF (%)	14 (46.7%)	8 (66.7%)	
TAF (%)	3 (10.0%)	0 (0.0%)	
ETV	6 (20.0%)	1 (8.3%)	
3TC/LdT	7 (23.3%)	3 (25.0%)	
Alcohol consumption (%)	6 (16.7%)	1 (12.5%)	1.000
Diabetes (%)	10 (33.3%)	2 (16.7%)	0.545
Previous hepatic decompensation			
Any	5 (16.7%)	7 (58.3%)	**0.020**
Ascites	4 (13.3%)	7 (58.3%)	**0.008**
Encephalopathy	0 (0.0%)	1 (8.3%)	0.631
Variceal bleeding	2 (6.7%)	1 (8.3%)	1.000
Varices (%)			0.414
Small	11 (36.7%)	2 (18.2%)	
Large	8 (26.7%)	5 (45.5%)	
Splenomegaly (%)	16 (53.3%)	11 (91.7%)	0.0051
HVPG ^1^, mmHg (IQR)	12 (9; 16)	22 (21; 22)	**0.023**
HVPG ≥ 16 mmHg (%)	3 (25.0%)	5 (100%)	**0.022**
LSM, kPa (IQR)	16.3 (10.0; 24.2)	47.7 (31.7; 75.0)	**<0.001**
LSM ≥ 25 kPa (%)	7 (23.3%)	12 (100%)	**<0.001**
CTP stage (%)			**<0.001**
A	28 (93.3%)	4 (33.3%)	
B	2 (6.7%)	8 (66.7%)	
MELD, points (IQR)	8 (7; 10)	11 (10; 12)	**0.002**
Albumin, g/L (IQR)	39.8 (37.5; 43.7)	34.6 (29.8; 36.3)	0.001
Bilirubin, mg/dL (IQR)	0.84 (0.59; 1.19)	1.31 (0.74; 2.20)	0.058
INR (IQR)	1.2 (1.1; 1.3)	1.3 (1.3; 1.5)	0.051
Creatinine, mg/dL	0.82 (0.74; 0.94)	0.72 (0.67; 0.99)	0.444
Sodium, mmol/L	139 (138; 141)	139 (135; 140)	0.207
Platelet count, G/L	146 (91; 195)	63 (48; 119)	**0.010**

^1^ HVPG available in *n* = 17 patients. Note: Bold fonts show statistically significant differences between groups. Abbreviations: FU = follow-up; IQR = interquartile range; BMI = body mass index; NA = nucleos(t)ide analogue; TDF = tenofovir disoproxile fumarate; TAF = tenofovir alafenamide; 3TC/LdT = lamivudine/telbivudine; HVPG = hepatic venous pressure gradient; LSM = liver stiffness measurement; CTP = Child-Turcotte-Pugh; MELD = model for end-stage liver disease; INR = international normalized ratio.

**Table 2:** Multivariate competing risk regression models with hepatic decompensation as outcome of interest and liver transplantation, diagnosis of hepatocellular carcinoma and non-liver-related death as competing risks. The parameter of interest (VCTE-LSM) is compared with the parameters that showed prognostic value in univariate analysis, i.e., (A) presence of ascites, (B) MELD and (C) albumin levels.

Patient Characteristic	Model A *			Model B			Model C		
	aSHR	95% CI	*p*	aSHR	95% CI	*p*	aSHR	95% CI	*p*
LSM, per kPA	1.03	1.01–1.06	**0.006**	1.04	1.02–1.06	**<0.001**	1.04	1.01–1.06	**0.002**
Ascites	3.43	0.93–12.63	0.064	-	-	-			
MELD, per point	-	-	-	1.14	1.05–1.23	**0.002**	-	-	-
Albumin, per g/L	-	-	-	-	-	-	0.93	0.84–1.03	0.150

* Model A was chosen by stepwise regression applying backward elimination including all four variables (see Supplementary Materials). Note: Bold fonts show statistically significant differences between groups. Abbreviations: aSHR = adjusted subdistribution hazard ratio; CI = confidence interval; LSM = liver stiffness measurement; MELD = model for end-stage liver disease score.

## Data Availability

Data can be obtained by contacting the corresponding author upon reasonable request.

## Supplementary Materials

The following supporting information can be downloaded at: https://www.mdpi.com/article/10.3390/jpm12020239/s1, Table S1: Comparison of patient characteristics of the overall cohort of patients under long-term chronic NA therapy (i.e., at the time of FU assessment); Table S2: Univariate competing risk regression models for the overall cohort with (further) hepatic decompensation as outcome of interest and diagnosis of hepatocellular carcinoma and non-liver-related death as competing risks; Table S3: Results of a stepwise competing risk regression with backward elimination aiming at selecting a final mode with minimal Akaike information criterion (AIC); Table S4: Univariate competing risk regression models for the subcohort of patients with compensated advanced chronic liver disease (cACLD) with first hepatic decompensation as outcome of interest and diagnosis of hepatocellular carcinoma and non-liver-related death as competing risks; Figure S1: Time-dependent receiver operating characteristic curves for the prediction of first hepatic decompensation in the subcohort of patients with compensated advanced chronic liver disease (cACLD) within three years of follow-up by HVPG (AUROC = 1.000 (95% CI 1.000–1.000); available in *n* = 11 patients), LSM (0.899 (0.757–1.000)), MELD (0.765 (0.533–0.996)), PLT (0.114 (0.000–0.277), inverse association) and albumin levels (0.084 (0.000–0.210), inverse association).
