# Supplementary material for: Long-Term Outcome of HBV-Infected Patients with Clinically Significant Portal Hypertension Achieving Viral Suppression
Source: J Pers Med. 2022 Feb 8;12(2):239. doi: 10.3390/jpm12020239 (PMC8880497; doi:10.3390/jpm12020239)
Supplement: Supplementary file 1 [file jpm-12-00239-s001.zip › jpm-1532593-supplementary.pdf]

Journal of Personalized Medicine

Supplementary Materials

Table S1. Comparison of patient characteristics of the overall cohort of patients under long-term chronic NA therapy (i.e., at the time of FU assessment).

Patient Characteristics	Overall Patient Cohort *n* = 42
Sex, male/female (% male)	33/9 (78.6%)
Age, years	48.3 (40.8; 56.4)
BMI, kg/m^2	26.4 (22.5; 30.4)
HBeAg positive (%)	6 (15.8%)
NA compound (%)	
TDF	22 (52.4%)
TAF	3 (7.1%)
ETV	7 (16.7%)
3TC/TLV	10 (23.8%)
Alcohol consumption (%)	9 (21.4%)
Diabetes (%)	12 (28.6%)
Previous hepatic decompensation (%)	
Any decompensation	12 (28.6%)
Ascites	11 (26.2%)
HE	1 (2.4%)
Variceal bleeding	3 (7.1%)
Varices (%)	
Small	13 (31.7%)
Large	13 (31.7%)
Splenomegaly (%)	27 (64.3%)
HVPG[1], mmHg	15 (10; 22)
HVPG ≥16 mmHg (%)	8 (47.1%)
LSM, kPa	22.5 (12.5; 41.0)
LSM ≥25 kPa (%)	19 (45.2%)
CTP stage (%)	
A	32 (66.2%)
B	10 (23.8%)
MELD, points	9 (7; 11)
Albumin, g/L	39.3 (35.7; 42,2)
Bilirubin, mg/dL	0.90 (0.67; 1.39)
INR	1.3 (1.1; 1.4)
Creatinine, mg/dL	0.79 (0.70; 0.97)
Sodium, mmol/L	139 (137; 141)
PLT, G/L	115 (75; 176)

[1] Available in n = 17 patients. Abbreviations: FU = follow-up, BMI = body mass index, NA = nucleos(t)ide analog, HE = hepatic encephalopathy, HVPG = hepatic venous pressure gradient, LSM = liver stiffness measurement, CTP = Child-Turcotte-Pugh score, INR = international normalized ratio, PLT = platelet count, TDF = tenofovir disoproxile fumarate, TAF = tenofovir alafenamide, ETV = entecavir, 3TC/TLV = lamivudine/telbivudine, MELD = model for end-stage liver disease score.

Table S2. Univariate competing risk regression models for the overall cohort with (further) hepatic decompensation as outcome of interest and diagnosis of hepatocellular carcinoma and non-liver-related death as competing risks.

Parameter	Univariate Model		
	**SHR**	**95% CI**	***p* Value**
LSM, per kPa	1.05	1.03–1.06	**<0.001**
Ascites	7.56	2.59–22.10	**<0.001**
MELD, per point	1.23	1.14–1.33	**<0.001**
Albumin, per g/L	0.87	0.80–0.95	**0.002**
PLT, per G/L	0.99	0.97–1.01	0.140

Abbreviations: LSM = liver stiffness measurement, SHR: subdistribution hazard ratio, 95% CI = 95% confidence interval, MELD = model for end-stage liver disease score; PLT = platelet count. Statistically significant values are bolded.

Table S3. Results of a stepwise competing risk regression with backward elimination aiming at selecting a final mode with minimal Akaike information criterion (AIC).

Parameter	Final Results of Stepwise Competing Risk Regression with Backward Elimination for Variable Selection	
	**Coefficient**	**Standard Error**
LSM, per kPa	0.033	0.012
Ascites	1.230	0.665
MELD, per point	-	-
Albumin, per g/L	-	-

Abbreviations: LSM = liver stiffness measurement, MELD = model for end-stage liver disease score.

Table S4. Univariate competing risk regression models for the subcohort of patients with compensated advanced chronic liver disease (cACLD) with first hepatic decompensation as outcome of interest and diagnosis of hepatocellular carcinoma and non-liver-related death as competing risks.

Parameter	Univariate Model		
	**SHR**	**95% CI**	***p* Value**
LSM, per kPa	1.06	1.04–1.09	**<0.001**
MELD, per point	1.22	0.96–1.53	0.100
Albumin, per g/L	0.858	0.78–0.95	**0.002**
PLT, per G/L	0.97	0.94–1.00	0.051

Abbreviations: LSM = liver stiffness measurement, SHR: subdistribution hazard ratio, 95% CI = 95% confidence interval, MELD = model for end-stage liver disease, PLT = platelet count. Statistically significant values are bolded.

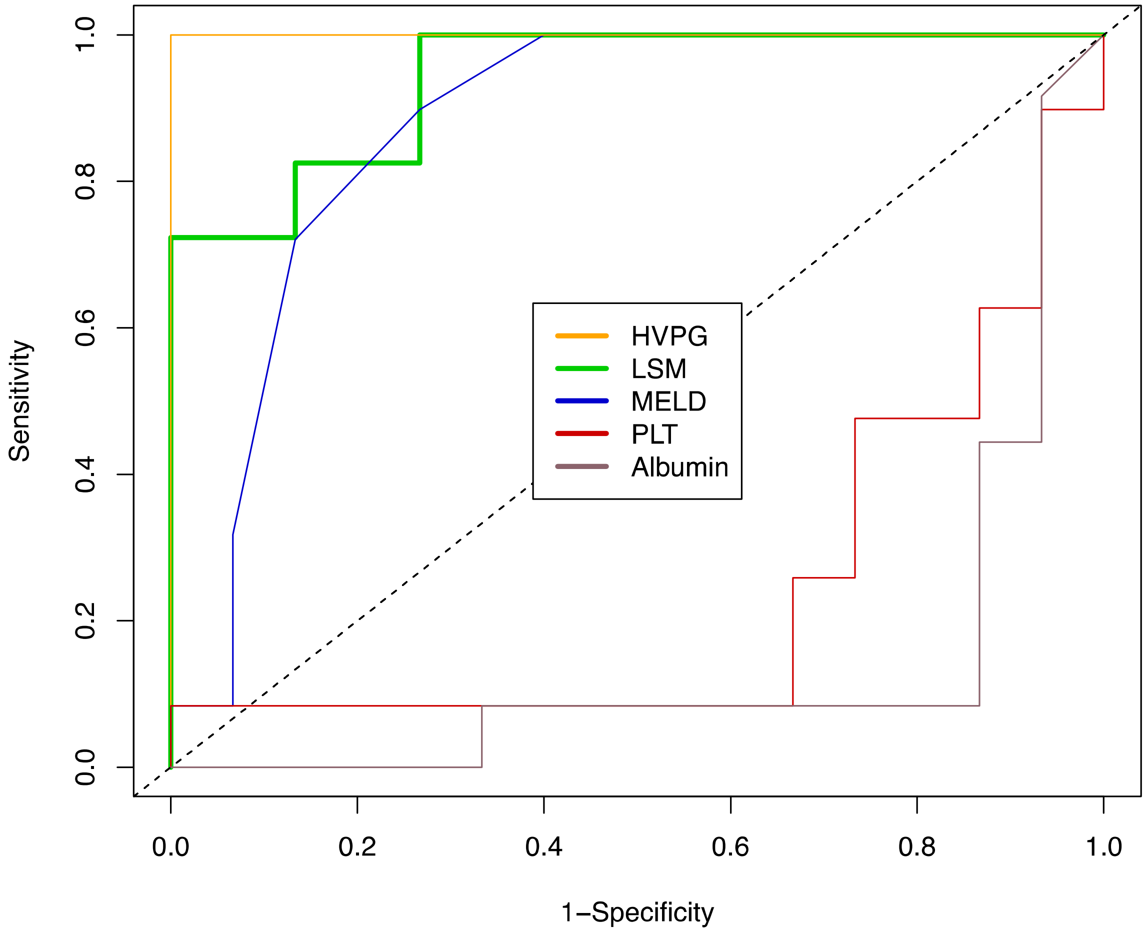

Figure S1. Time-dependent receiver operating characteristic curves for the prediction of first hepatic decompensation in the subcohort of patients with compensated advanced chronic liver disease (cACLD) within three years of follow-up by HVPG (AUROC = 1.000 (95% CI 1.000–1.000); available in *n* = 11 patients), LSM (0.899 (0.757–1.000)), MELD (0.765 (0.533–0.996)), PLT (0.114 (0.000–0.277), inverse association) and albumin levels (0.084 (0.000–0.210), inverse association). Abbreviations: HVPG = hepatic venous pressure gradient; AUROC = area under the receiver operating characteristics curve; CI = confidence interval; LSM = liver stiffness measurement; MELD = model for end-stage liver disease score; PLT = platelet count.
